# Interaction between NSCLC Cells, CD8^+^ T-Cells and Immune Checkpoint Inhibitors Potentiates Coagulation and Promotes Metabolic Remodeling—New Cues on CAT-VTE

**DOI:** 10.3390/cells13040305

**Published:** 2024-02-07

**Authors:** Catarina Freitas-Dias, Filipe Gonçalves, Filipa Martins, Isabel Lemos, Luís G. Gonçalves, Jacinta Serpa

**Affiliations:** 1 iNOVA4Health, NOVA Medical School, Faculdade de Ciências Médicas, NMS, FCM, Universidade NOVA de Lisboa, Campo dos Mártires da Pátria, 130, 1169-056 Lisboa, Portugal; cfdias.bio@gmail.com (C.F.-D.); fggoncalves@ipolisboa.min-saude.pt (F.G.); filipa.martins@nms.unl.pt (F.M.); a2022507@nms.unl.pt (I.L.); 2 Instituto Português de Oncologia de Lisboa Francisco Gentil (IPOLFG), Rua Prof Lima Basto, 1099-023 Lisboa, Portugal; 3 Faculdade de Ciências, FCUL, Universidade de Lisboa, Campo Grande, 130, 1169-056 Lisboa, Portugal; 4 Instituto de Tecnologia Química e Biológica António Xavier (ITQB NOVA), Avenida da República (EAN), 2780-157 Oeiras, Portugal; lgafeira@itqb.unl.pt

**Keywords:** cancer-associated thrombosis (CAT), venous thromboembolism (VTE), immune checkpoint inhibitors (ICIs), non-small cell lung cancer (NSCLC), metabolic remodeling, tissue factor (TF)

## Abstract

Background: Cancer-associated thrombosis (CAT) and venous thromboembolism (VTE) are frequent cancer-related complications associated with high mortality; thus, this urges the identification of predictive markers. Immune checkpoint inhibitors (ICIs) used in cancer immunotherapy allow T-cell activation against cancer cells. Retrospective studies showed increased VTE following ICI administration in some patients. Non-small cell lung cancer (NSCLC) patients are at high risk of thrombosis and thus, the adoption of immunotherapy, as a first-line treatment, seems to be associated with coagulation-fibrinolysis derangement. Methods: We pharmacologically modulated NSCLC cell lines in co-culture with CD8^+^ T-cells (TCD8^+^) and myeloid-derived suppressor cells (MDSCs), isolated from healthy blood donors. The effects of ICIs Nivolumab and Ipilimumab on NSCLC cell death were assessed by annexin V and propidium iodide (PI) flow cytometry analysis. The potential procoagulant properties were analyzed by in vitro clotting assays and enzyme-linked immunosorbent assays (ELISAs). The metabolic remodeling induced by the ICIs was explored by ^1^H nuclear magnetic resonance (NMR) spectroscopy. Results: Flow cytometry analysis showed that TCD8^+^ and ICIs increase cell death in H292 and PC-9 cells but not in A549 cells. Conditioned media from NSCLC cells exposed to TCD8^+^ and ICI induced in vitro platelet aggregation. In A549, Podoplanin (PDPN) levels increased with Nivolumab. In H292, ICIs increased PDPN levels in the absence of TCD8^+^. In PC-9, Ipilimumab decreased PDPN levels, this effect being rescued by TCD8^+^. MDSCs did not interfere with the effect of TCD8^+^ in the production of TF or PDPN in any NSCLC cell lines. The exometabolome showed a metabolic remodeling in NSCLC cells upon exposure to TCD8^+^ and ICIs. Conclusions: This study provides some insights into the interplay of immune cells, ICIs and cancer cells influencing the coagulation status. ICIs are important promoters of coagulation, benefiting from TCD8^+^ mediation. The exometabolome analysis highlighted the relevance of acetate, pyruvate, glycine, glutamine, valine, leucine and isoleucine as biomarkers. Further investigation is needed to validate this finding in a cohort of NSCLC patients.

## 1. Introduction

Lung cancer is a prevalent, quickly metastasizing and highly aggressive malignancy [1]. According to the International Agency for Research on Cancer, in 2020, lung cancer had an estimated incidence of 2.2 million cases, with 1.8 million registered fatalities worldwide [2]. Histologically, lung cancer is commonly divided into two major groups: small cell lung cancer (SCLC) and non-small cell lung cancer (NSCLC) [3]. NSCLC is more prevalent [4], representing about 85% of lung cancers [5,6], and SCLC composes the remaining 15% [5,7]. 

One of the most recent emerging hallmarks of cancer is metabolic remodeling, which is receiving increased attention globally as a result of its critical role in the development, progression and therapy resistance in cancer [8,9]. Cancer cells require metabolic remodeling in order to survive and adjust to the conditions in the tumor microenvironment (TME), therefore the metabolic rewiring to meet the energetic and synthetic requirements is crucial to support the increased proliferation [10,11]. The metabolic network is highly dependent on cancer cells’ interaction with other cells in the TME, including immune effector cells. 

Immune cells within the TME can lack the ability to attack the tumor and additionally contribute to tumor growth and disease progression [12]. A key element of the TME is often the tumor-infiltrating lymphocytes (TILs), which may comprise varying ratios of CD4^+^ and CD8^+^ T-cells (TCD8^+^) [12]. The cytotoxic reactions of TILs are directed towards capturing and eliminating malignant cells, being the TCD8^+^, the primary immune killer cells [13,14]. Another component of the TME is the myeloid-derived suppressor cell (MDSC) population [15], which is recruited by the tumor. MDSCs are a heterogeneous cell group [16] that suppresses T-cell proliferation and activity [12,17] through different mechanisms, including arginase-1 and nitric oxide secretion and reactive oxygen species (ROS) production [16,18].

T-cells play an important part in immunotherapy since they oversee cell-mediated immunity [19]. For instance, the discovery of cytotoxic TCD8^+^ as potent effectors of the adaptive anti-tumor immune response has resulted in their widespread usage in both model systems and therapies [20,21,22]. The immunotherapeutic approach rests on the interaction of the TCD8^+^ with cancer cells through specific immune regulatory mechanisms. Malignant cells escape recognition and attack from TCD8^+^ by expressing inhibitory receptors normally found on healthy cells [23]. The discovery of this ability has led to the immune checkpoint blockade treatment, which causes TCD8^+^ activation and enhances malignant cell clearance [13,19,24]. The blockade strategies usually act upon two immune checkpoint pathways: programmed cell death 1 (PD1) and cytotoxic T-lymphocyte antigen 4 (CTLA4) [14,22,24].

Advanced NSCLC has a very poor outcome, with a mean expected survival of about 7 months [25]. Immune checkpoint inhibitor (ICI) therapy for NSCLC seems to increase the patients’ survival to a range between 8 and 15 months [26,27,28]. Nonetheless, the increase in the immune response induced by ICIs can cause secondary effects related to an unbalanced homeostasis of the systemic immune system [29]. Postow et al. (2018) proposed that the immune system may have a bigger impact on these manifestations than the tumor itself [24].

Thrombotic complications in cancer patients are common [30,31] and are denominated cancer-associated thrombosis (CAT), but their risk can be influenced by the therapy applied [32,33,34]. Some studies analyzed the relationship between thromboembolic events and ICI treatment in NSCLC patients, showing, for the first time, that the risk of thrombosis is not negligible [33,35,36,37,38,39]. Further research is needed to discover precise mechanisms underlying the interaction of hemostasis, immunological response and cancer in the context of ICI therapy [29]. To enhance early detection and promote the implementation of primary thromboprophylaxis, which is not routinely recommended for NSCLC patients because of bleeding risk, it is vitally necessary to understand the underlying processes of ICI-caused disorders of the coagulation–fibrinolysis system and their impact on thrombosis and bleeding [29,30].

Thrombosis-associated metabolomics is understudied, but it may offer substantial potential for biomarker identification, especially given the current surge in interest in this topic [40]. Some metabolites have been described in the literature to be altered in thrombosis and related conditions in vivo, such as glycine, glutamine, valine, leucine, isoleucine and alanine [41,42,43].

The aim of this study was to explore in vitro the dynamics between NSCLC cells, TCD8^+^ and ICI exposure and evaluate its impact on coagulation. Inherently, we also aimed to identify potential prothrombotic biomarkers that could be eventually used in the clinics to aid CAT prediction and the need for prophylaxis.

## 2. Materials and Methods

### 2.1. Immune Cell Isolation

CD8^+^ T-cells (TCD8^+^) and MDSCs were obtained from peripheral blood, collected under the consenting donation of healthy donors from Serviço de Imuno-Hemoterapia at Instituto Português de Oncologia de Lisboa Francisco Gentil (IPOLFG) (IPOLFG-Ethical committee UIC-1349). Peripheral blood mononuclear cells (PBMCs) from blood samples were isolated using Ficoll-Paque (17144003, Amersham Biosciences, Cytiva, Marlborough, MA, USA), according to the manufacturer’s indications. Each biological replicate resulted from a pool of peripheral blood collected from 3 randomized healthy donors in order to keep individual variability.

TCD8^+^ were isolated from PBMCs using the CD8^+^ T-Cell Isolation Kit—human (130-096-495, Miltenyi Biotec, San Jose, CA, USA), according to the manufacturer’s indications. CD8^+^ cells were recovered from the PBMC through an exclusion cell separation system of antibodies and magnetic beads, in which the fraction rich in CD8^+^ T-cells was obtained. MDSCs were obtained from PBMCs by inducing in vitro derivation, according to the protocol described by Choi et al., 2022 [44]. PBMCs were cultured in plates with Roswell Park Memorial Institute 1640 medium (RPMI: 21870076, Gibco, ThermoFisher Scientific, Waltham, MA, USA) supplemented with 10% fetal bovine serum (FBS, S 0615, Merck, Rahway, NJ, USA), 1% Antibiotic-Antimycotic (AA, P06-07300, PAN Biotech), 50 μg/mL Gentamicin (15750-060, Gibco, Life Technologies, Waltham, MA, USA) and 1% L-Glutamine (25-005-CI, Corning, New York, NY, USA). Additionally, to induce MDSC derivation, the culture medium was supplemented with 20 ng/mL interleukin-6 (IL-6: I1395-10UG, Merck, Sigma-Aldrich, St. Louis, MO, USA) and 20 ng/mL granulocyte-macrophage colony-stimulating factor (GM-CSF: H5666-10UG, Merck, Sigma-Aldrich). Cells were incubated for 6 days at 37 °C, and 5% CO_2_ and medium was refreshed every 3 days. After 6 days of induction, derived MDSCs were magnetically sorted to CD33^+^ and CD11b^+^ cells using the following kits: CD33 MicroBeads—human (130-045-501, Miltenyi Biotec) and CD11b MicroBeads—human (130-049-601, Miltenyi Biotec), according to the manufacturer’s indications.

### 2.2. Cell Lines and Culture Conditions

Human adenocarcinoma cell line A549 (CCL-185^TM^, ATCC) and mucoepidermoid carcinoma cell line H292 (CRL-1848^TM^, ATCC) were purchased from the American Type Culture Collection (ATCC, Manassas, VA, USA). Adenocarcinoma cell line PC-9 (90071810, ECACC) was purchased from the European Collection of Authenticated Cell Cultures (ECACC, Porton Down, Salisbury, UK). 

The NSCLC cell lines used have different *EGFR* and *KRAS* mutational profiles. A549 cells are *EGFR* WT (wild-type) and *KRAS* c.34G > A (p.Gly12Ser). H292 are *EGFR* WT and *KRAS* WT. PC-9 cells are *KRAS* WT and *EGFR* exon 19 deletion (Ex19Del, Glu746-Ala750 deletion). 

H292 and PC-9 cells were cultured in Dulbecco’s Modified Eagle’s Medium (DMEM) (11965092, Gibco, ThermoFisher Scientific) and A549 in Dulbecco’s Modified Eagle’s Medium/Nutrient Mixture F-12 Ham medium (DMEM/F-12: 11320033, Gibco, ThermoFisher Scientific). Both culture media were supplemented with 10% FBS, 1% AA and 50 μg/mL Gentamicin. Cells were maintained at 37 °C in a humidified environment at 5% CO_2_. Cells were cultured until an optical confluence of 75–100%, and detachment was performed with 0.05% trypsin-EDTA 1× (25300-054, Invitrogen, Waltham, MA, USA). 

For the experimental conditions (applied in the day following the co-culture establishment), cells were exposed to Nivolumab (16 µg/mL, OPDIVO, anti-PD1) or Ipilimumab (48 µg/mL, YERVOY, anti-CTLA4) for 48 h. The Nivolumab and Ipilimumab concentrations were chosen in accordance with the posology indications from the European Medicines Agency (EMA), specifically 1 mg/Kg for OPDIVO and 3 mg/Kg for YERVOY [45,46], and using as reference a male adult with 80 kg and 5 L of blood. The conditions were tested with or without TCD8^+^. 

The co-cultures with TCD8^+^ were established indirectly, using 1 μm pore TransWell inserts (932 10 02, cellQART), or directly, by using three ratios (1:1, 2:1 and 3:1) of TCD8^+^ to NSCLC cell number. For the enzyme-linked immunosorbent assays, the NSCLC cells were co-cultured with TCD8^+^ and MDSCs.

### 2.3. Cell Death Analysis

In order to evaluate the capacity of TCD8^+^ to induce cancer cell death influenced or not by ICIs, cell death analysis was assessed by flow cytometry; thus, after the experimental conditions, the supernatants (conditioned media) were collected. The cells were harvested with trypsin-EDTA 1× and centrifuged at 150× *g* for 2 min, along with cells in the supernatant. The supernatant was discarded, and the pellet washed with 200 µL PBS 1 × −0.1% (*v*/*w*) bovine serum albumin (BSA) and centrifuged at 150× *g* for 2 min. Cells were suspended with 0.5 μL Annexin V-fluorescein (FITC) (640906, BioLegend, Koblenz, Germany) in annexin V binding buffer 1 × (10 mM HEPES (pH 7.4; 391333, Millipore, New York, NY, USA), 140 mM sodium chloride (NaCl; 106404, Merck) and 2.5 mM calcium chloride (CaCl_2_; 449709, Sigma-Aldrich) and incubated at room temperature, in the dark for 15 min. After incubation, samples were washed with PBS 1 × −0.1% (*v*/*w*) BSA and centrifuged at 150× *g* for 2 min. The cell pellets were resuspended in 100 μL of annexin V binding buffer 1× and stained with 1.25 μL propidium iodide (PI, 50 μg/mL, P4170, Sigma-Aldrich). Additionally, an anti-CD45-APC antibody was used to detect TCD8^+^ (555485, Becton Dickinson, Heidelberg, Germany) in order to exclude putative contaminations with immune cells from co-culture. The samples were analyzed by flow cytometry (BD Accuri^TM^ C6 flow cytometer—Becton Dickinson) using the BD Accuri C6 Software v1.0.34.1. The experiments were performed in biological triplicates.

### 2.4. Spectrophotometric Determination of Platelet Aggregation

Platelet-rich plasma (PRP) from healthy donors was gently assigned by Serviço de Imuno-Hemoterapia at Instituto Português de Oncologia de Lisboa Francisco Gentil (IPOLFG) (IPOLFG-Ethical committee UIC-1349). The platelet suspensions were centrifuged at 2650× *g* for 3 min. The precipitated platelets were carefully resuspended in the conditioned media and plated in 96-well plates at a concentration of 5 × 10^5^ platelets/µL (5 × 10^7^ platelets/well). For positive controls, platelets were resuspended in unconditioned medium and epinephrin was added to induce aggregation. Upon 5, 15 and 30 min of exposure to the conditioned media, the absorbance was measured at an interval of one minute at 595 nm with gentle shaking in an iMark Microplate Absorbance Reader (1681130, Bio-Rad, Hercules, CA, USA). The relative aggregation for each experimental condition was calculated using the following formula:

$$\%\;\mathrm{Aggregation}=\frac{{\mathrm{Abs}}_{\;\mathrm{sample}}}{{\mathrm{Abs}}_{\;\mathrm{Pos}\;\mathrm{CTL}}}\times 100$$

In which Abs _sample_ is the absorbance measured in the samples (platelets in conditioned culture media) and Abs _Pos CTL_ is the absorbance measured in the epinephrin positive control.

### 2.5. Enzyme-Linked ImmunoSorbent Assay (ELISA) Detection of Tissue Factor and Podoplanin

Detection of extracellular concentration of procoagulant proteins Tissue factor (TF) and Podoplanin (PDPN) was made by enzyme-linked immunosorbent assays (ELISAs). TF is associated with hypercoagulation in cancer patients [47]. PDPN is associated with thrombotic disease [48], and it seems to contribute to cancer progression as a mediator of cancer promoting inflammation [49]. Extracellular TF was detected and quantified using the Human Tissue Factor ELISA kit (ab220653, abcam, Cambridge, UK). Extracellular PDPN was detected and quantified using the Human Podoplanin ELISA kit (EH375RB: Invitrogen, ThermoFisher Scientific). Prior to the ELISAs, the conditional culture media were concentrated in ultrafiltration centrifuge tubes (UFC8010, Merck, Sigma-Aldrich) at 3220× *g* for 6 min. After concentrating the samples, the assays were performed following the manufacturer’s protocols. The original concentrations of TF and PDPN in the tested samples were calculated by extrapolating the results measured to the original sample volume.

### 2.6. Nuclear Magnetic Resonance (NMR) Spectroscopy

Nuclear magnetic resonance (NMR) spectroscopy was performed for extracellular metabolites of NSCLC cell lines. Conditioned culture media (supernatants of the established co-cultures) were collected and stored at −80 °C. A volume of 30 µL of 0.4% (*v/v*) sodium azide and 30 μL of 2.2 mM 3-(trimethylsilyl) propionic 2,2,3,3-d4 acid (TSP), both in deuterated water (D_2_O), were added to 540 μL of supernatants. TSP was used as an internal reference for quantification and chemical shift. ^1^H-NMR spectra were acquired in an Ultrashied^TM^ 500 Plus spectrometer (Bruker, Oeiras, Portugal) with a TCI-Z probe at 25 °C. Spectra were acquired with a *noesypr1d* pulse program, free induction decay (FID) size = 48k points, 128 scans, with 4 s of acquisition time and 10 ms of mixing time. Spectra were processed using TopSpin 4.1 software (Bruker), and the metabolite assignments and quantifications were made by resorting to spectral databases as Chenomx NMR Suite 8.11 and Human Metabolome (HMDB).

### 2.7. Statistical Analysis

Statistical analysis was performed using GraphPad Prism 8.0 software (https://www.graphpad.com). Sample data were presented as the mean (normal distribution) ± SD. All assays were performed with three biological replicates per treatment (N = 3). Comparisons between data from each group were statistically analyzed by a two-tailed unpaired Student’s *t*-test, while multiple comparisons were performed using one-way ANOVA with Tukey’s test. For cell death analysis and platelet aggregation assays, a two-way ANOVA with Tukey’s or Sidak’s test were used. Differences between experimental conditions were considered statistically significant for *p* < 0.05. Multivariate statistical analysis of ^1^H-NMR data was performed on MetaboAnalyst 5.0 (https://www.metaboanalyst.ca/; assessed on 17 January 2024) using metabolite concentrations as inputs that were scaled using Pareto-scaling. The heatmaps were created with the following parameters: Euclidean distance and Ward’s clustering method. Enrichment plots representing multivariate pathways analysis of the extracellular levels of the different metabolites were created for each cell line using GraphPad Prism 8.0 software.

## 3. Results

### 3.1. Validation of the Model of TCD8^+^–Cancer with ICI

The purpose of the following results was to validate the model of TCD8^+^–cancer cell interaction in order to conduct the primary studies described below.

**Figure 1:**
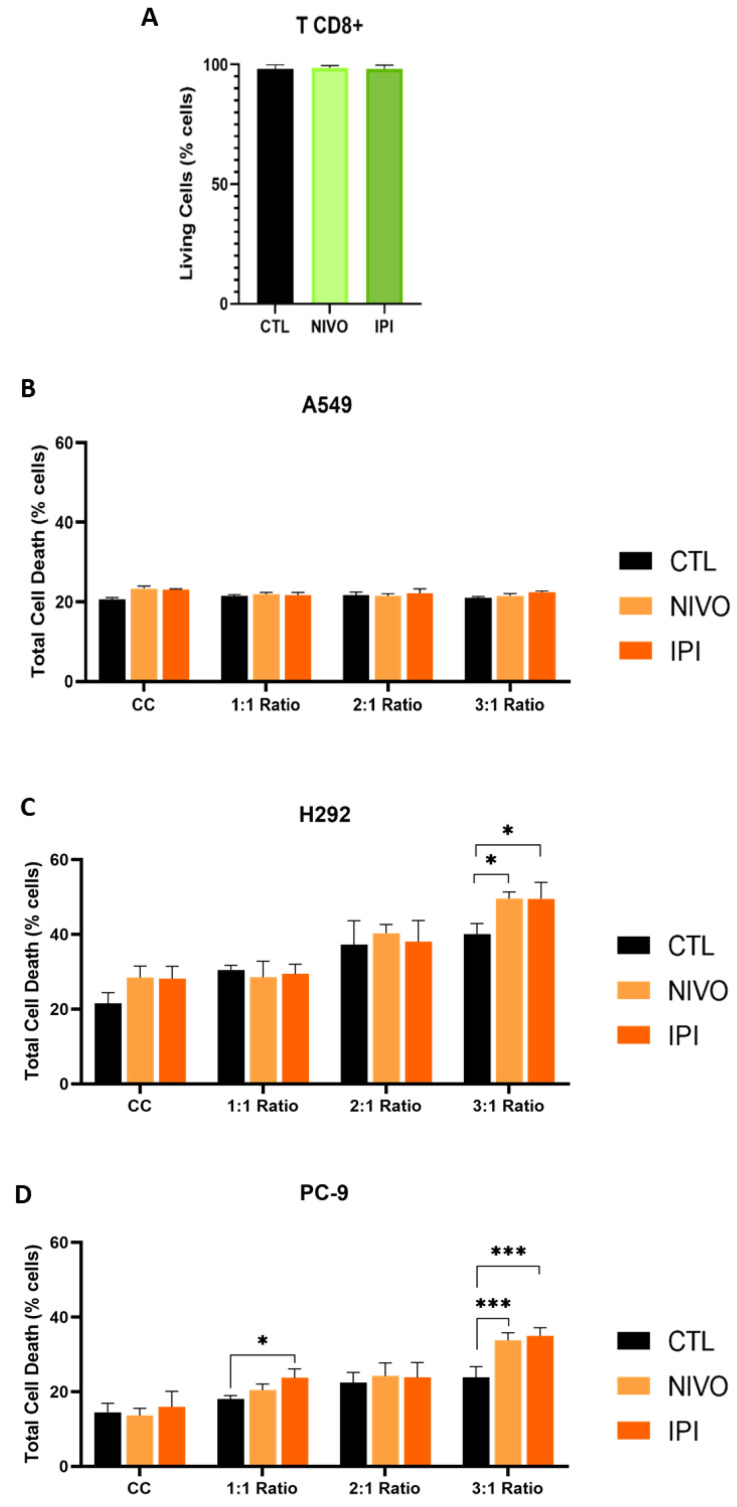
Nivolumab and Ipilimumab affect viability of H292 and PC-9 cells in the presence of TCD8^+^. NSCLC cells were co-cultured with TCD8^+^ in different TCD8^+^: cancer cell ratios (1:1, 2:1 and 3:1). The cells were cultured in control (CTL), Nivolumab (NIVO) and Ipilimumab (IPI) conditions. Cell death was evaluated by flow cytometry (N = 3). (**A**) Nivolumab and Ipilimumab did not affect viability of TCD8^+^. (**B**) A549 cell death was not affected by the experimental conditions. In cell lines H292 (**C**) and PC-9 (**D**), Nivolumab and Ipilimumab caused a significant increase in cell death in 3:1 ratio co-culture. Results are represented as mean ± SD. * *p* < 0.05, *** *p* < 0.001. For (**A**), unpaired *t*-tests were used. For (**B**–**D**), two-way ANOVAs were used with Tukey’s test. CC: cancer cells (monoculture).

Cell death analysis was performed to evaluate the effect of the ICIs on cancer cell viability when in the presence of TCD8^+^ and upon Nivolumab or Ipilimumab treatment during 48 h (Figure 1). It was observed that neither Nivolumab (*p* = 0.6278) nor Ipilimumab (*p* = 0.9622) altered the viability of TCD8^+^ (Figure 1A). Regarding NSCLC cells, A549 cells did not show cell death differences in co-culture with TCD8^+^ or treatment with ICIs (Figure 1B). The control conditions at 2:1 and 3:1 ratios induced significant H292 and PC-9 cell death (Table 1 and Table 2 and Supplementary Figure S1), suggesting that TCD8^+^ alone were able to affect the viability of cancer cells. The H292 cell line showed a significant increase in cell death upon co-culture in a 3:1 ratio, with and without ICIs (Figure 1C and Supplementary Figure S1). Accordantly, in the PC-9 cell line, a significant increase in cell death was verified for the conditions at 3:1 co-cultures (Figure 1D and Supplementary Figure S1).

### 3.2. Co-Culture with TCD8^+^ and Treatment with Nivolumab or Ipilimumab Increase Platelet Aggregation In Vitro

**Figure 2:**
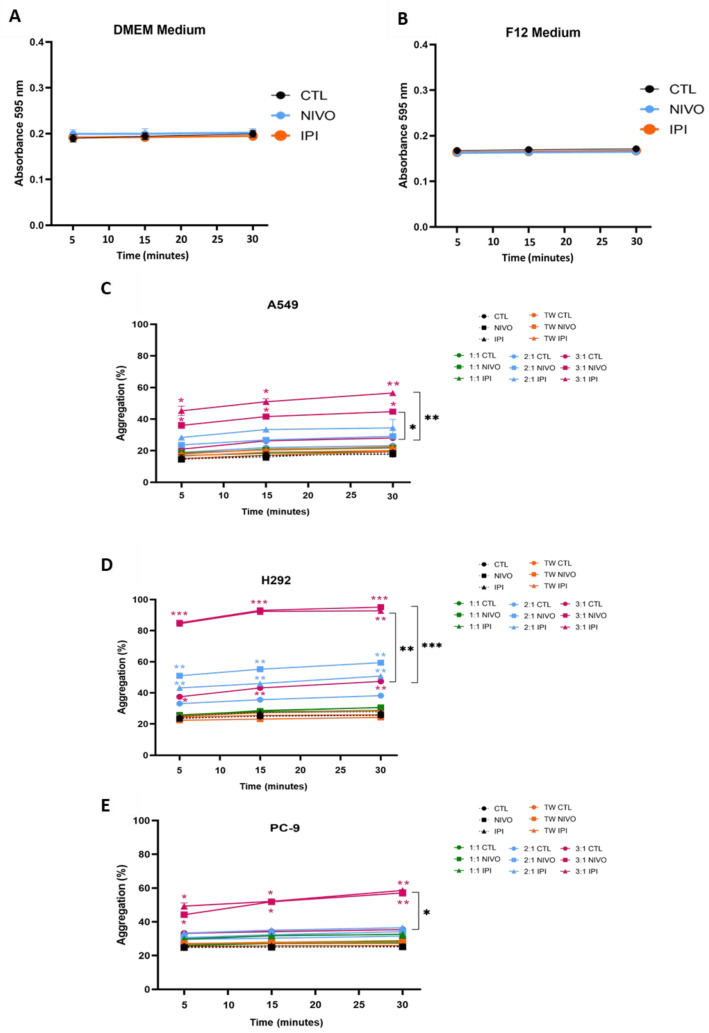
Co-culture with TCD8^+^ and treatment with Nivolumab or Ipilimumab increase platelet aggregation in vitro. Absorbance of culture medium was measured in control (CTL), Nivolumab (NIVO) and Ipilimumab (IPI) conditions. The presence of Nivolumab and Ipilimumab does not alter absorbance of DMEM (**A**) or DMEM/F-12 (**B**) media. Platelets were treated with the cultures’ supernatants for (**C**–**E**) (N = 3). (**C**) Within the conditions from A549 cells, platelet aggregation increased with direct co-cultures with TCD8^+^ and when exposed to Nivolumab or Ipilimumab. The 3:1 ratios with Nivolumab and with Ipilimumab induced the highest effect in A549 cells. (**D**) Media from 2:1 and 3:1 ratio conditions on H292 cell cultures caused significant platelet aggregation. (**E**) From the conditions in PC-9 cell cultures, platelet aggregation significantly increased with direct co-cultures with TCD8^+^ at a 3:1 ratio exposed to Nivolumab or Ipilimumab. The aggregation levels are relative to aggregation caused by epinephrin, used as a positive control. Results are represented as mean ± SD. * *p* < 0.05, ** *p* < 0.01, *** *p* < 0.001. Colorful significances compare the colored condition with the assay control. Further statistical data are available in Supplementary Tables S1–S3. Two-way ANOVAs were used with Sidak’s test. TW: TransWell conditions (indirect co-cultures).

We evaluated the impact of soluble molecules from the cancer cells’ environment on platelet aggregation by a spectrophotometric assay. We first verified that neither Nivolumab nor Ipilimumab affected the media absorbance at 595 nm (Figure 2A,B). Then, for A549 samples, no effect on platelet aggregation was observed with the conditioned media from monocultures treated with ICIs (Supplementary Table S1), as well as from the control conditions in co-cultures (both direct and indirect) (Figure 2C). 

The conditioned media from 3:1 (TCD8^+^: NSCLC cells) co-cultures treated with Nivolumab and Ipilimumab promoted platelet aggregation in vitro for conditioned media from all NSCLC cell lines (Figure 2C–E). However, no effect on platelet aggregation was observed with the conditioned media from ICI-treated monocultures, which means in the absence of TCD8^+^ (Figure 2C–E). In addition, for all NSCLC cell lines, a clear distinction between direct and indirect co-cultures regarding aggregation capability of the media was observed. While direct co-cultures promoted high aggregation indexes, the indirect co-cultures did not (Figure 2C–E). These results suggest the need for cell-to-cell interactions between TCD8^+^ and cancer cells to influence thrombogenesis. 

Within the A549 setting, the 3:1 (TCD8^+^: A549) cultures treated with Ipilimumab showed the highest aggregation percentage (Figure 2C). It is important to point out that platelets with 3:1 TCD8^+^:H292 media achieved over 90% aggregation (Figure 2D), while the highest values seen in the other cell lines were about 60% (Figure 2C,E). In H292, the media from 2:1 (TCD8^+^: H292) ICIs exposure conditions increased platelet aggregation compared to control conditions (Figure 2D, Supplementary Table S2). In PC-9 cells, only the 3:1 (TCD8^+^: PC-9) culture media with ICIs were able to increase aggregation (Figure 2E). Thus, a high content of TCD8^+^ is related to an increased aggregation of platelets, and ICIs are required to enhance the effect of TCD8^+^ on platelet aggregation, indicating an immune modulation of CAT.

### 3.3. TF and PDPN Protein Levels Are Differently Affected by Treatment with ICIs in NSCLC Cell Lines

**Figure 3:**
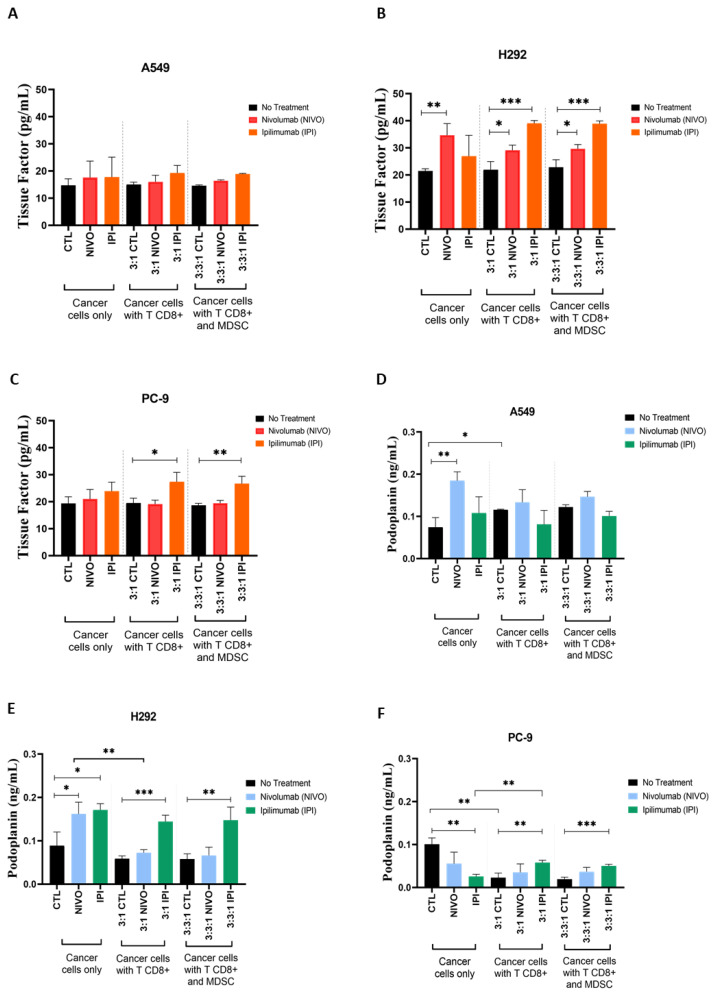
Tissue factor (TF) and Podoplanin (PDPN) protein levels are differently affected by treatment with ICIs in NSCLC cell lines. Culture supernatants’ analysis of TF and PDPN proteins by enzyme-linked immunosorbent assay (ELISA) (N = 3). (**A**) In cultures with A549 cells, TF levels were only slightly higher with Nivolumab and Ipilimumab exposure (compared to the respective controls). (**B**) In cultures with H292 cells, TF levels were increased in co-cultures with TCD8^+^ that were treated with both ICIs. (**C**) In cultures with PC-9 cells, treatment with Ipilimumab increased TF levels in the presence of TCD8^+^. (**D**) In cultures with A549 cells, PDPN levels were only slightly decreased in the presence of TCD8^+^ and the ICI (compared to monocultures). (**E**) With H292 cells, co-cultures with TCD8^+^ that were treated with ICIs showed an increase in PDPN protein. (**F**) In cultures with PC-9 cells, TCD8^+^ presence induced a small decrease in PDPN with Nivolumab, but an increase was seen with Ipilimumab. Results are shown as mean ± SD. * *p* < 0.05, ** *p* < 0.01, *** *p* < 0.001. Unpaired *t*-tests were used.

Since the 3:1 ratio (TCD8^+^:NSCLC cells) was the one presenting higher aggregation levels (Figure 2), this was the ratio selected to be evaluated for extracellular levels of TF and PDPN. TF in media from A549 cultures presented only a trend to increase with ICIs, comparing to the respective controls (Figure 3A). Regarding H292 (Figure 3B), Nivolumab induced a significant increase in TF levels in the culture media, and there was also a higher TF concentration in the presence of both TCD8^+^ and the ICIs (Figure 3B). In PC-9 cultures, TF levels showed an increase with the co-culture 3:1 (TCD8^+^:PC-9) condition treated with Ipilimumab (Figure 3C).

Concerning PDPN, A549 cells with Nivolumab presented increased PDPN levels, an effect that is lost when co-cultured with TCD8^+^ (Figure 3D). Intriguingly, H292 and PC-9 media in control cultures without TCD8^+^ contained significantly less PDPN than the monoculture’s control (Figure 3E,F). H292 showed increased levels of PDPN with Nivolumab and Ipilimumab, but in the presence of TCD8^+^, only treatment with Ipilimumab remained significant (Figure 3E). H292 cells co-cultured with TCD8^+^ and treated with Nivolumab showed significantly lower levels of PDPN than in the absence of TCD8^+^ (Figure 3E). PC-9 treated with Ipilimumab presented decreased levels of PDPN in the media (Figure 3F), but this effect was significantly rescued by TCD8^+^, with the PDPN levels being higher than in the control (Figure 3F). 

To evaluate if MDSC exposure would rescue the TF and PDPN levels induced by the TCD8^+^, supernatants from NSCLC cells co-cultured with TCD8^+^ and MDSC exposed to ICIs were also analyzed. For the three cell lines, media from NSCLC cells with TCD8^+^ and MDSCs did not show differences in these proteins’ levels when compared to NSCLC cultured with TCD8^+^ (Figure 3A–F). Therefore, even though a consistent pattern was not verified in the tested conditions, TF should be further studied as a potential marker for CAT. Additionally, MDSCs do not affect the levels of TF or PDPN, not even through the modulation of TCD8^+^ action.

### 3.4. TCD8^+^ and ICIs Impact NSCLC and Promote Metabolic Remodeling

**Figure 4:**
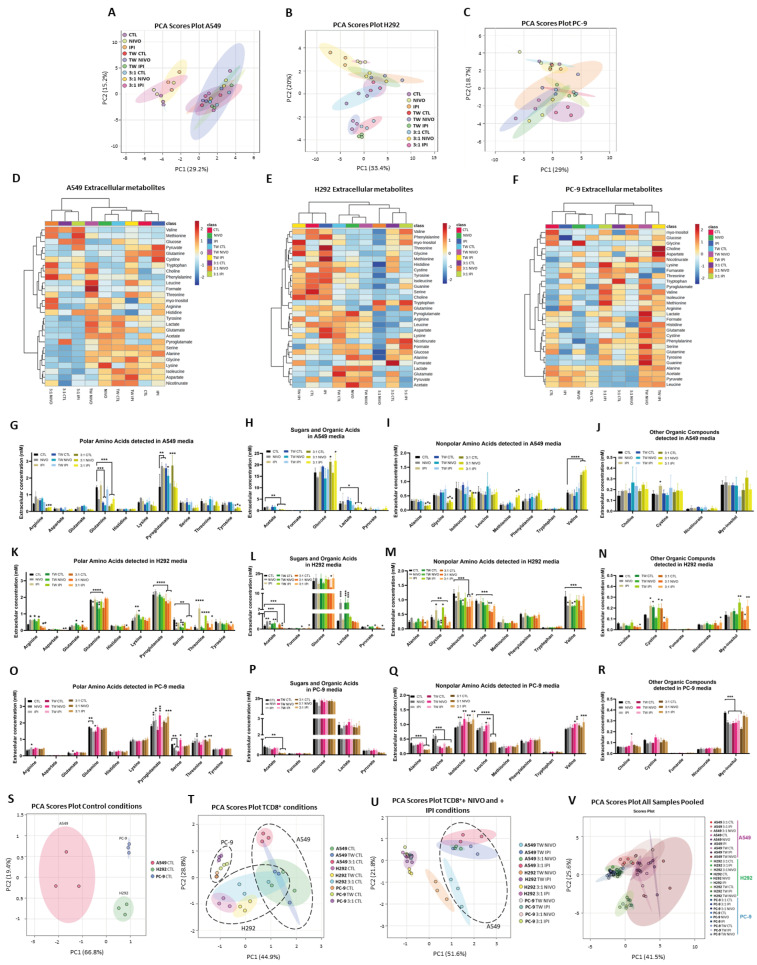
Exometabolome of NSCLC cells is affected by TCD8^+^ and ICI treatment. The cell culture media were analyzed by nuclear magnetic resonance (NMR) in order to study the exometabolome of the TME and detect metabolic variations between CTL, NIVO, IPI, 3:1 CTL, 3:1 NIVO and 3:1 IPI conditions (N = 3). A principal component analysis (PCA) was conducted to classify the samples, and the scores plots illustrate clustering patterns from the exometabolome profiles of samples from A549 (**A**), H292 (**B**) and PC-9 (**C**) cell lines. Heatmaps showing the concentrations of extracellular metabolites in A549 (**D**), H292 (**E**) and PC-9 (**F**) cell lines. Concentration data in the heatmaps represent the average values obtained from the replicates of the experimental conditions. Heatmaps color codes represent the relative fold change of each metabolite between classes, being red and blue colors increased or decreased levels, respectively. Euclidean distance measure and the Ward cluster algorithm were used for the heatmaps analysis, using *MetaboloAnalyst* 5.0 software. The levels of the 27 metabolites identified in A549 cultures media were organized into polar amino acids (**G**), sugar and organic acids (**H**), nonpolar amino acids (**I**) and other organic compounds (**J**). The levels of the 28 metabolites identified in H292 cultures media were organized into polar amino acids (**K**), sugar and organic acids (**L**), nonpolar amino acids (**M**) and other organic compounds (**N**). The levels of the 28 metabolites identified in PC-9 cultures media were organized into polar amino acids (**O**), sugar and organic acids (**P**), nonpolar amino acids (**Q**) and other organic compounds (**R**). A principal component analysis (PCA) was conducted to classify the samples, and the scores plots illustrate clustering patterns considering the levels of acetate, pyruvate, glutamine, glycine, valine, leucine and isoleucine in samples from A549 (**S**), H292 (**T**) and PC-9 (**U**) cell lines individually and pooled together (**V**). Represented in the graphics (**G**–**R**) is the statistical analysis, for each metabolite detected, of the different treatment conditions compared to the control condition. * *p* < 0.05, ** *p* < 0.01, *** *p* < 0.001, **** *p* < 0.0001. One-way ANOVA was used, followed by Tukey’s test. TW: TransWell conditions (indirect co-cultures).

Because several metabolites have been shown in the literature to be altered in thrombosis and other related complications in vivo, the effect of ICIs and TCD8^+^ on the exometabolome of all NSCLC cell lines was investigated by analyzing the conditioned culture media by ^1^H-NMR spectroscopy. Principal component analysis (PCA) was performed using the determined metabolites’ concentrations to assess metabolic patterns dependent on TCD8^+^ or the ICI exposure (Supplementary Table S4). The PCA showed a clustering of A549 samples by experimental conditions: the monoculture conditions (control, Nivolumab and Ipilimumab) cluster together and are separated by the first principal component (PC1) from all the co-cultures with TCD8^+^ (Figure 4A). In H292 and PC-9 culture media, the PCA showed more diversity between the different co-cultures (Figure 4B,C). Interestingly, H292 metabolism seems to be more susceptible to exposure to Nivolumab and Ipilimumab without TCD8^+^, since the control cluster does not overlap with Nivolumab and Ipilimumab (Figure 4B), as seen for A549 and PC-9. Nevertheless, the heatmaps regarding univariate analysis of the extracellular metabolites’ concentrations propose that the exometabolome of the TME may present alterations due to the exposure to TCD8^+^ and/or ICIs (Figure 4D–F). More detailed data regarding the replicates’ concentrations are available in Supplementary Figure S2. These effects bring up the possibility that CTLA4 and PD1 are important mediators of cancer metabolic remodeling.

### 3.5. The Heterogeneity of the Exometabolome of the Different NSCLC Cell Lines

On the extracellular metabolites of A549 cells cultured with TCD8^+^ and exposed to ICIs, it was observed that most of the significant effects were induced by the TCD8^+^ presence, mainly in direct co-cultures. It was seen that extracellular acetate, alanine, arginine, glutamine, glycine, lactate and tyrosine concentrations decreased with the direct presence of TCD8^+^ (control and ICI treatments) (Figure 4G–J). Conversely, levels of glucose and valine were increased in the same conditions (Figure 4H). As seen previously in the PCA (Figure 4B), in H292, a higher impact of the ICI treatments was visible in the detected metabolites. In H292 media, we verified a decrease in acetate and pyruvate levels with Ipilimumab treatments in both the presence and absence of TCD8^+^ (Figure 4K–N). As for arginine levels, there was a decrease seen upon ICI treatments in TCD8^+^ presence, and extracellular threonine concentration was significantly higher with Ipilimumab treatments in any culture type applied (Figure 4K). It was also found that Nivolumab influenced the levels of some metabolites. Opposite to Ipilimumab, acetate tended to increase with Nivolumab treatments (Figure 4L). In addition, extracellular glycine, isoleucine and valine tended to decrease with Nivolumab, in the presence and absence of TCD8^+^ (Figure 4M). Regarding PC-9 media, extracellular acetate, alanine, and leucine decreased with the direct presence of TCD8^+^ independently of the treatment (Figure 4O–R). Leucine levels were also affected by the indirect exposure of TCD8^+^ since an increase was verified in ICI-treated cultures (Figure 4Q). Extracellular glycine was also influenced by TCD8^+^ but regardless of the co-culture approach: a significant reduction was seen for both direct and indirect presence of TCD8^+^ (Figure 4O). As verified previously for the platelet aggregation assay, also in this metabolic analysis, it appears to be an important distinction between direct and indirect co-cultures. Many of the metabolite alterations seen with TCD8^+^ presence are associated with their direct contact to the NSCLC cells (Figure 4G–R), suggesting, again, a modulation through the interaction of the two cell types. Lastly, Ipilimumab treatments increased isoleucine and valine extracellular levels (Figure 4Q). Overall, CTLA4 and PDL1 play a role in the control of cancer cell metabolism.

The TME exometabolome showed that acetate, pyruvate, glutamine, glycine, valine, leucine and isoleucine were indicative of increased coagulative conditions, thus, considering these compounds, PCA analysis was performed evaluating all cell lines together. The control conditions of A459, H292 and PC-9 samples were evaluated, and PCA showed independent clusters according to the NSCLC cell line (Figure 4S). The TCD8^+^:NSCLC co-cultures presented more similarities between the NSCLC cell line samples (Figure 4T). Upon ICI treatment, H292 and PC-9 samples were clustered together, indicating increased similarities of the TME exometabolome due to ICI exposure (Figure 4U). When all the samples from the three cell lines were pooled together, the samples tended to separate more by cell line than by ICI exposure (Figure 4V). Therefore, a common profile is achieved in NSCLC under the action of ICIs.

## 4. Discussion

The existence of a connection between cancer and thrombosis due to a tumor-driven hypercoagulable state has long been described [50,51]. Although the particular processes involved are not entirely known [50], tumor-related hypercoagulability remains one of the most important indicators predicting overall survival [52]. CAT is common among cancer patients, yet the thrombotic risk is highly variable among tumor types [35,36,37,38], and NSCLC patients seem to be at high risk for thromboembolic events, mainly VTE [53]. At the same time, the ICI treatment, in monotherapy or in association with chemotherapy, seems to increase CAT [33,54]. It has also been previously proposed that an ICI-induced systemic proinflammatory state may aggravate the prothrombotic status by activating coagulation and platelets while hindering fibrinolysis [29,55].

In this study, we proposed that the dynamics between the ICIs (Nivolumab and Ipilimumab) and the NSCLC cells with TCD8^+^ influence, to some extent, the environmental coagulation status. To our knowledge, this is the first study that investigates these dynamics in vitro. Importantly, we reported that the combination of ICIs and TCD8^+^ not only increases cancer cell death but also seems to induce platelet aggregation. Currently, in vitro studies evaluating the cytotoxic effects of ICIs over NSCLC cells are scarce, thus emphasizing the importance of the in vitro assessments we carried out. Importantly, we cannot discard the occurrence of non-self-reactions preconized by TCD8^+^ cells against NSCLC cells, with no direct relation to PD1 and CTLA4. However, we can postulate if ICIs affect TCD8^+^-mediated cancer cell death. 

As expected, H292 and PC-9 exhibited elevated death levels when co-cultured with increased quantities of TCD8^+^ cells and treated with ICIs (Figure 1). Interestingly, in H292 and PC-9, cell death does increase in co-cultures without ICI exposure, implying that TCD8^+^ alone can affect cancer cell viability. Interestingly, as the number of TCD8^+^ increased, a synergistic effect of cell death with ICI was observed (Figure 1). This agrees with data showing that Nivolumab is more successful in tumors with substantial TCD8^+^ infiltration [56]. Therefore, here, we developed a useful model for studying the interaction of TCD8^+^ cell cytotoxicity and thrombogenesis.

However, the viability of A549 cells was not affected by any experimental condition tested (Figure 1), and this result is in accordance with another study testing Nivolumab in this cell line [57]. The lack of responsiveness could be due to the low levels of PD1, PDL1 and CD80/CD86 detected in A549 cells [58,59,60], although these cells express high levels of CTLA4 [61]. This cell line is mutated for *KRAS*, and for this molecular type of adenocarcinomas, only the ones expressing high levels of PDL1 are good responders to ICI therapy [62,63]. Moreover, the ICI treatment might not result in viability changes if the immune response is suppressed by an untargeted mechanism [64]. In fact, A549 is an adenocarcinoma cell line, and this type of NSCLC is recognized for its therapy resistance [65]. 

Given that thromboembolic events within the CAT spectrum have been associated with ICI therapy [33,54], with the intent of understanding if the TME had procoagulant properties, the impact on platelets of soluble molecules of the culture media was evaluated. Regarding procoagulant properties of the TME, there was no effect of TCD8^+^ alone on platelet aggregation in A549 samples since none of the co-culture control conditions were significant (Figure 2C). Platelet aggregation was enhanced in ICI-treated co-cultures with the largest proportion of TCD8^+^ (conditions 3:1 co-cultures with NIVO and 3:1 co-cultures with IPI) (Figure 2C). TCD8^+^ were able to trigger some aggregation in H292 and PC-9 samples, but only at high concentrations, which increased even more with the ICI treatments (Figure 2D,E). For the three NSCLC cell lines, there was a significant difference in media aggregation capabilities between direct and indirect co-cultures. Direct co-cultures could reach high aggregation indexes, but indirect co-cultures did not cause significant aggregation (Figure 2C–E). These findings support the hypothesis of a necessity for cell-to-cell contacts between TCD8^+^ and cancer cells to drive thrombogenesis. Despite the fact that the extracellular media of the three cell lines displayed varied aggregation patterns, the 3:1 co-cultures with NIVO and 3:1 co-cultures with IPI conditions resulted in the highest aggregation percentage across them (Figure 2). As a result, the combination of ICIs and a high number of TCD8^+^ cells appears to trigger cancer-related coagulation in NSCLC.

Platelet aggregation was previously considered to be a simple process, but it has become clear that this mechanism is far more complex, being today depicted as a multistep adhesion mechanism that involves several different molecule interactions [66,67]. The in vitro platelet aggregation assay performed was an extremely simplified model to study the cohesion. The platelets isolated from plasma were treated with the cultures’ conditioned medium without any further supplements. This is critical since platelet aggregation generally requires three components: an agonist, a plasma protein and adhesion molecules [68]. In this case, we used the conditioned culture media as potential activating stimuli. Stimulus activation of integrin αIIbβ3 (present on the platelet’s surface) is essential to bind the soluble protein [69] and consequently bind two neighboring platelets. According to the findings, the conditioned medium was sufficient to operate as an agonist and offer soluble molecules that allow platelets to bridge together. In vivo, fibrinogen is the most prevalent plasma protein responsible for platelet-to-platelet bonding [70]. Fibrinogen would be secreted into the medium for it to be present in the analyzed samples. Some studies support this concept, citing the potential of cancer cells, as well as platelets, to generate and release fibrinogen [70,71,72,73]. It could be interesting to evaluate fibrinogen presence in the samples in further research. Because the coagulation cascade normally occurs extracellularly and most coagulation factors are present in the blood but not in culture media, the effects observed in vitro are unlikely to be the genuine coagulation cascade. Nonetheless, these may represent the cascade’s latest steps as a result of the presence of fibrinogen. Perhaps studying this behavior in more complex models would be beneficial; regardless it was sensitive enough to detect differences.

The analysis of TF in conditioned media showed that under control conditions of monocultured NSCLC cells, all cell lines released TF into the medium (Figure 3A–C). This was expected given previous research on circulating TF in cancer [74,75]. In contrast, such a disparity in the effects of TCD8^+^ and ICIs on TF levels among the cell lines was unexpected. The TCD8^+^ and ICIs had no significant effect on TF levels in A549 cells, although a trend to increase TF with ICIs was observed (Figure 3A). The A549 cell line holds characteristics that make it less responsive to the tested conditions, and maybe an intrinsic resistance to therapy may underlie this behavior. In H292, Nivolumab administration enhanced TF levels with and without TCD8^+^, but Ipilimumab only increased TF when TCD8^+^ were present (Figure 3B). This suggests that, unlike Nivolumab, Ipilimumab’s effect must be mediated by TCD8^+^. In PC-9 medium, TF levels were only higher when TCD8^+^ and Ipilimumab were combined (Figure 3C). These findings imply that if the same dynamic happens in vivo, ICI treatment may induce an increase in circulating TF and, as a result, an activation of the coagulation protease cascade in NSCLC patients. According to the literature, the TME can alter TF expression in cancer cells through the introduction of stressful situations such as hypoxia, inflammation and interactions with other cells [76,77]. Tumor-associated factors may regulate TF expression, resulting in differences between cancer types and subtypes [78], which is consistent with the findings observed in the present study. It is also worth noting that research in this area is continuous, and the link between TF expression and cancer behavior appears to be complex [79]. 

Analogously to TF, we observed that there was PDPN release under control conditions of monocultured NSCLC cells (Figure 3D–F). For the A549 cell line (Figure 3D), Nivolumab alone increased extracellular PDPN levels, while the combination of TCD8^+^ and the ICIs resulted in just a modest reduction in PDPN, which was not regarded as significant. Both Nivolumab and Ipilimumab alone enhance PDPN concentration in H292 cultures, but Nivolumab’s effects subsided in the presence of TCD8^+^ (Figure 3E). PC-9 was the cell line with the lowest PDPN concentrations in the medium, and Ipilimumab alone reduced PDPN levels (Figure 3F). Although TCD8^+^ alone reduced PDPN levels, the combination of TCD8^+^ and Ipilimumab substantially increased PDPN secretion (Figure 3F). While we expected PDPN levels to rise after treatment with TCD8^+^ and ICIs, they decreased in some cases instead. Since PDPN has other roles independent of coagulation, this observation can be related to the PDPN inhibitory effect on TCD8^+^ [80]. Therefore, in situations requiring TCD8^+^ action, PDPN expression may be shut down. As a result, the discrepancies between and within cell lines are intriguing, possibly illustrating an unanticipated complex control system for PDPN, underlay by its different functions. 

Since MDSCs are known to suppress T-cell proliferation and activity [12,17] and are an important element of the TME [15], we consider that the incorporation of this population in our study is relevant. To determine if MDSC exposure would reinstate the TF and PDPN levels induced by the TCD8^+^, the co-cultures were analyzed with the addition of MDSCs. Here, in vitro, we observed that the co-culture of MDSCs with TCD8^+^:NSCLC co-cultures did not abrogate the effects caused by TCD8^+^ in TF and PDPN levels. The results of cancer cells with TCD8^+^ and MDSCs did not differ statistically from those of cancer cells with TCD8^+^ but no MDSCs (Figure 3). Nonetheless, this does not mean that MDSCs are irrelevant in this context, since it has been shown that increased TF impacts MDSCs by promoting their recruitment, which is linked to cancer progression [81]. More research is needed to clarify the role of MDSCs in CAT. We provide indications that TCD8^+^ may moderate some effects in the dynamic evaluated in this study; consequently, these effects could be altered in the presence of MDSCs if they act as TCD8^+^ suppressors. Therefore, it is important to evaluate the MDSCs’ impact on additional outcomes in subsequent work, such as cell viability and the metabolic alterations.

While malignant tumors exhibit metabolic adaptability, some metabolites have been described to be altered in pro-coagulant contexts [41,42,43]. Therefore, to better understand the metabolic remodeling that occurs during the interaction of cancer cells with TCD8^+^ and ICIs that may lead to coagulation increase, the exometabolome was analyzed. Among the alterations observed for A549 cultures, we can highlight the decrease in acetate, arginine and glutamine in the presence of TCD8^+^ (Figure 4G,H). Lower levels of acetate may be related to its use as a substrate to fuel growth or fatty acids synthesis induced by the TCD8^+^, while lower levels of arginine may be linked to apoptosis induction in the cancer cells [82]. As such, arginine uptake and metabolism by TILs is used for the release of nitric acid [83]. Arginine is also implicated in cancer cell proliferation, since many tumor cells, including A549 cells, are arginine auxotrophic [84]. Glutamine is an important substrate for several metabolic processes, acting as a nitrogen and carbon donor [85], and may be consumed by both cancer cells and TCD8^+^. For H292, the uptake of acetate and pyruvate (acetyl-CoA precursors) by cancer cells after Ipilimumab treatment (Figure 4L) may indicate an increase in the TCA cycle or fatty acid production [86,87]. Ipilimumab treatment also considerably increased extracellular threonine levels (Figure 4K). Threonine is an amino acid that is essential to produce certain proteins, particularly threonine-rich proteins such as mucins [88], which have been suggested to contribute to tumor invasiveness [89]. H292 cells are mucus-secreting cells derived from a mucoepidermoid carcinoma [90], producing mucins with threonine-rich domains [91,92]. Taking this into consideration, Ipilimumab may affect mucin synthesis by H292 cells via changes in threonine consumption. In PC-9, acetate levels decreased with TCD8^+^ exposure (Figure 4P). It has previously been described that acetate induces epigenetic changes in TCD8^+^ cells, such as histone acetylation, which increases IFN-γ transcription and subsequent release, hence enhancing tumor clearance [93]. In fact, conditions with lower amounts of acetate (Figure 4P) were associated with higher levels of cell death in PC-9 cells (Figure 1D). Glycine also decreased with TCD8^+^, independently of direct or indirect co-cultures, indicating the existence of an influence that is not dependent on cell-to-cell contact between TCD8^+^ and cancer cells. In addition to participating in one-carbon metabolism, de novo purine synthesis and creatine and glutathione synthesis, glycine metabolism is important for porphyrin synthesis [94,95,96,97]. Since porphyrins are necessary components of hemoglobin, the alterations observed could alter the systemic hemoglobin availability. Hemoglobin < 10 g/dL is a variable in Khorana score to determine the thrombotic risk [98], therefore, alterations in glycine availability can impact CAT. To ensure that anticoagulant treatment is handled efficiently and safely, it is critical to determine the precise influence of glycine levels in hemoglobin and this connection in vivo. The results additionally suggest that Ipilimumab and TCD8^+^ may have influence over the pathways of valine, leucine and isoleucine biosynthesis and degradation in PC-9 cells.

As indicated by the PCAs and metabolite plots (Figure 4), we propose that TCD8^+^ and ICIs have a great influence on the in vitro exometabolome. Overall, some of the exometabolome changes observed may be connected to the presence and nutrient competition of TCD8^+^ cells in the culture since T lymphocytes rely on external nutrition availability [99]. Nonetheless, and intriguingly, we discovered that Nivolumab and Ipilimumab produced changes even without TCD8^+^ mediation in certain cases. While the major mechanism of ICIs is immune activation, their effect can be exerted on cancer cells expressing the antigens. NSCLC cells are described as intrinsically expressing CTLA4, which modulates PDL1 expression [61], and here, we show these antigens regulate the metabolic adaptation, which is a pro-survival cellular phenomenon. Consequently, it is worthwhile to investigate the extent to which ICI-induced metabolic remodeling may contribute to cancer development and, in some cases, therapeutic resistance mechanisms.

Thrombosis-associated metabolomics is not often investigated, but it may hold significant promise for biomarker discovery given the rising interest in this field in recent years [40]. Interestingly, metabolic profiles including acetate, pyruvate, glutamine, glycine, valine, leucine and isoleucine were found to be distinctive of conditions inducing higher platelet aggregation rates and TF production, which involved the co-cultures of TCD8^+^ with H292 and PC-9 cell lines and the exposure to Ipilimumab (Figure 4U). We found evidence in the literature that some of these metabolites are altered and associated with thrombosis and related conditions in vivo. In detail, changes in glycine serum levels have been linked to cardiovascular disease [41], and we observed glycine is decreased in 3:1 co-cultures with ICIs in both H292 and PC-9. Obi et al. (2016) proposed glutamine as a possible elevated metabolite in VTE [42]. Cao et al. (2018) investigated the metabolic fingerprints of deep vein thrombosis (DVT) and discovered that its incidence is related to greater blood levels of glutamine, valine and leucine, as well as changes in the valine, leucine and isoleucine biosynthesis pathway [43]. Interestingly, we observed in 3:1 H292 and PC-9 co-cultures with ICIs increased levels of glutamine, valine and isoleucine, while leucine levels increased in 3:1 H292 co-cultures with ICIs. In a rat model, the authors also discovered that decreased alanine levels, as we observed in our setting, are linked to DVT [43].

## 5. Conclusions

This study sheds more light on the pro-coagulant effect of ICIs, highlighted by the increased platelet aggregation. This seems to result from a cooperative effect between TCD8^+^, NSCLC cells and the ICI treatments. Additionally, we reinforce that TF could underlie the platelet aggregation and the pro-coagulant effect of ICIs in NSCLC patients. The metabolic remodeling associated with the pro-coagulant phenotype highlighted acetate, pyruvate, glutamine, glycine, valine, leucine and isoleucine as promising biomarkers. Therefore, the monitoring of TF levels and metabolic profiles in peripheral blood of patients conditioned with ICIs, during follow-up, can be a good opportunity to anticipate CAT-VTE and improve the clinical management, allowing a timely intervention. Overall, this reinforces the need for characterization of patients’ biological samples to validate the profiles defined in this study, and maybe in the future these profiles can serve as a tool for stratification of patients for CAT-VTE prophylaxis.

## Figures and Tables

**Table 1:** Statistical analysis cell death evaluation for H292 by flow cytometry. Data analysis performed with two-way ANOVA followed by Tukey’s test. Results that were considered significant: * *p* < 0.05, ** *p* < 0.01, *** *p* < 0.001, **** *p* < 0.0001; “ns” means not significant.

Compared Samples		*p*-Value	Summary
CTL	NIVO	0.0773	ns
	IPI	0.0928	ns
	1:1 CTL	0.0329	*
	2:1 CTL	0.0001	***
	3:1 CTL	<0.0001	****
NIVO	1:1 NIVO	>0.9999	ns
	2:1 NIVO	0.0034	**
	3:1 NIVO	<0.0001	****
IPI	1:1 IPI	0.9758	ns
	2:1 IPI	0.0159	*
	3:1 IPI	<0.0001	****
1:1 CTL	1:1 NIVO	0.7994	ns
	1:1 IPI	0.9365	ns
2:1 CTL	2:1 NIVO	0.5824	ns
	2:1 IPI	0.963	ns
3:1 CTL	3:1 NIVO	0.0126	*
	3:1 IPI	0.013	*

**Table 2:** Statistical analysis cell death evaluation for PC-9 by flow cytometry. Data analysis performed with two-way ANOVA followed by Tukey’s test. Results that were considered significant: * *p* < 0.05, ** *p* < 0.01, *** *p* < 0.001, **** *p* < 0.0001; “ns” means not significant.

Compared Samples		*p*-Value	Summary
CTL	NIVO	0.919	ns
	IPI	0.7908	ns
	1:1 CTL	0.3983	ns
	2:1 CTL	0.008	**
	3:1 CTL	0.0018	**
NIVO	1:1 NIVO	0.026	*
	2:1 NIVO	0.0004	***
	3:1 NIVO	<0.0001	****
IPI	1:1 IPI	0.01	**
	2:1 IPI	0.0089	**
	3:1 IPI	<0.0001	****
1:1 CTL	1:1 NIVO	0.5437	ns
	1:1 IPI	0.0468	*
2:1 CTL	2:1 NIVO	0.7035	ns
	2:1 IPI	0.8146	ns
3:1 CTL	3:1 NIVO	0.0005	***
	3:1 IPI	0.0001	***

## Data Availability

Metabolomics data are available in a workbench metabolomics repository with the DataTrack ID 4469 (https://www.metabolomicsworkbench.org/).

## Supplementary Materials

The supporting information can be downloaded at: https://www.mdpi.com/article/10.3390/cells13040305/s1.
