# Supplementary material for: Interaction between NSCLC Cells, CD8+ T-Cells and Immune Checkpoint Inhibitors Potentiates Coagulation and Promotes Metabolic Remodeling—New Cues on CAT-VTE
Source: Cells. 2024 Feb 7;13(4):305. doi: 10.3390/cells13040305 (PMC10886748; doi:10.3390/cells13040305)
Supplement: Supplementary file 1 [file cells-13-00305-s001.zip › cells-2832653-supplementary.pdf]

Supplementary materials

Supplementary Table S1. Statistical analysis of condition-based evaluation of platelet aggregation with conditioned media from A549 cultures. Data analysis performed with Two-way ANOVA followed by Sidak's test. Were considered significant: *$p<0.05$, **$p<0.01$, ***$p<0.001$, ****$p<0.0001$.

Compared Samples		At 5 minutes		At 15 minutes		At 30 minutes	
		p-value	Summary	*p-value*	Summary	*p-value*	Summary
CTL	NIVO	>0.9999	ns	>0.9999	ns	>0.9999	ns
	IPI	>0.9999	ns	>0.9999	ns	>0.9999	ns
	TW CTL	>0.9999	ns	>0.9999	ns	>0.9999	ns
	1:1 CTL	>0.9999	ns	>0.9999	ns	>0.9999	ns
	2:1 CTL	0.8587	ns	0.9820	ns	0.4942	ns
	3:1 CTL	0.0117	*	0.2994	ns	0.4010	ns
NIVO	TW NIVO	0.9977	ns	0.2821	ns	0.0559	ns
	1:1 NIVO	0.2021	ns	0.0364	*	0.0014	**
	2:1 NIVO	0.0186	*	0.0228	*	0.5385	ns
	3:1 NIVO	0.0609	ns	0.1188	ns	<0.0001	****
IPI	TW IPI	>0.9999	ns	0.8042	ns	0.9348	ns
	1:1 IPI	0.6142	ns	0.0516	ns	0.2017	ns
	2:1 IPI	0.0367	*	0.0114	*	0.0094	**
	3:1 IPI	0.0027	**	0.0056	**	0.0002	***
TW CTL	TW NIVO	0.9968	ns	0.0621	ns	0.3096	ns
	TW IPI	0.5520	ns	0.2282	ns	0.2352	ns
1:1 CTL	1:1 NIVO	0.2018	ns	0.1584	ns	0.0040	**
	1:1 IPI	0.0485	*	0.3247	ns	0.0117	*
2:1 CTL	2:1 NIVO	0.0628	ns	0.6838	ns	0.7893	ns
	2:1 IPI	0.0062	**	0.0681	ns	0.0059	**
3:1 CTL	3:1 NIVO	0.1443	ns	0.2340	ns	0.0033	**
	3:1 IPI	0.0945	ns	0.0679	ns	0.0389	*

Supplementary Table S2. Statistical analysis of condition-based evaluation of platelet aggregation with conditioned media from H292 cultures. Data analysis performed with Two-way ANOVA followed by Sidak's test. Were considered significant: **p<0.05*, ***p<0.01*, ****p<0.001*, *****p<0.0001*.

Compared Samples		At 5 minutes		At 15 minutes		At 30 minutes	
		p-value	Summary	*p-value*	Summary	*p-value*	Summary
CTL	NIVO	>0.9999	ns	>0.9999	ns	>0.9999	ns
	IPI	>0.9999	ns	0.9928	ns	0.6265	ns
	TW CTL	>0.9999	ns	>0.9999	ns	>0.9999	ns
	1:1 CTL	0.9860	ns	0.9708	ns	0.7379	ns
	2:1 CTL	0.0055	**	0.0262	*	0.1105	ns
	3:1 CTL	0.0316	*	0.0034	**	0.0179	*
NIVO	TW NIVO	>0.9999	ns	>0.9999	ns	0.8044	ns
	1:1 NIVO	0.7112	ns	0.9865	ns	0.1094	ns
	2:1 NIVO	0.0019	**	0.0024	**	0.0001	***
	3:1 NIVO	<0.0001	****	0.0047	**	<0.0001	****
IPI	TW IPI	>0.9999	ns	>0.9999	ns	>0.9999	ns
	1:1 IPI	>0.9999	ns	>0.9999	ns	0.9497	ns
	2:1 IPI	0.0023	**	0.2437	ns	0.0399	*
	3:1 IPI	0.0002	***	0.0336	*	0.0118	*
TW CTL	TW NIVO	0.0282	*	0.5149	ns	0.9919	ns
	TW IPI	>0.9999	ns	0.9993	ns	0.9010	ns
1:1 CTL	1:1 NIVO	>0.9999	ns	>0.9999	ns	0.9994	ns
	1:1 IPI	>0.9999	ns	>0.9999	ns	0.9996	ns
2:1 CTL	2:1 NIVO	0.0132	*	0.0188	*	0.0082	**
	2:1 IPI	0.0552	ns	0.1707	ns	0.0256	*
3:1 CTL	3:1 NIVO	0.0021	**	0.0039	**	0.0003	**
	3:1 IPI	0.0002	***	0.0045	**	0.0002	***

Supplementary Table S3. Statistical analysis of condition-based evaluation of platelet aggregation with conditioned media from PC-9 cultures. Data analysis performed with Two-way ANOVA followed by Sidak's test. Were considered significant: *$p<0.05$, **$p<0.01$, ***$p<0.001$, ****$p<0.0001$.

Compared Samples		At 5 minutes		At 15 minutes		At 30 minutes	
		p-value	Summary	*p-value*	Summary	*p-value*	Summary
CTL	NIVO	>0.9999	ns	>0.9999	ns	>0.9999	ns
	IPI	>0.9999	ns	>0.9999	ns	>0.9999	ns
	TW CTL	>0.9999	ns	>0.9999	ns	>0.9999	ns
	1:1 CTL	>0.9999	ns	0.8406	ns	0.5384	ns
	2:1 CTL	0.6754	ns	0.0920	ns	0.0492	*
	3:1 CTL	0.2074	ns	0.0634	ns	0.0033	**
NIVO	TW NIVO	0.7584	ns	0.4597	ns	0.0054	**
	1:1 NIVO	0.7858	ns	0.2163	ns	0.3142	ns
	2:1 NIVO	0.0072	**	0.0020	**	0.0008	***
	3:1 NIVO	0.1197	ns	0.0125	*	0.1165	ns
IPI	TW IPI	>0.9999	ns	0.8393	ns	0.4470	ns
	1:1 IPI	0.9752	ns	0.0510	ns	0.0131	*
	2:1 IPI	0.2329	ns	0.1147	ns	0.0018	**
	3:1 IPI	0.0402	*	0.0002	***	0.0147	*
TW CTL	TW NIVO	0.9956	ns	0.9988	ns	0.9977	ns
	TW IPI	0.9998	ns	0.9865	ns	0.9807	ns
1:1 CTL	1:1 NIVO	>0.9999	ns	>0.9999	ns	0.9466	ns
	1:1 IPI	0.9863	ns	0.1257	ns	0.0550	ns
2:1 CTL	2:1 NIVO	>0.9999	ns	0.9597	ns	0.0364	*
	2:1 IPI	0.7818	ns	0.5062	ns	0.0182	*
3:1 CTL	3:1 NIVO	0.1905	ns	0.0760	ns	0.1767	ns
	3:1 IPI	0.1774	ns	0.0061	*	0.0453	*

Supplementary Table S4. Metabolites identified in ^{1}H-NMR spectra in the NSCLC cultures established. Growth media spectra of A549, H292 and PC-9 cultures were acquired with a noesypr1d pulse program.

Metabolites	A549	H292	PC-9
Acetate	X	X	X
Alanine	X	X	X
Arginine	X	X	X
Aspartate	X	X	X
Choline	X	X	X
Cystine	X	X	X
Formate	X	X	X
Fumarate		X	X
Glucose	X	X	X
Glutamate	X	X	X
Glutamine	X	X	X
Glycine	X	X	X
Histidine	X	X	X
Isoleucine	X	X	X
Lactate	X	X	X
Leucine	X	X	X
Lysine	X	X	X
Methionine	X	X	X
Myo-inositol	X	X	X
Nicotinurate	X	X	X
Phenylalanine	X	X	X
Pyroglutamate	X	X	X
Pyruvate	X	X	X
Serine	X	X	X
Threonine	X	X	X
Tryptophan	X	X	X
Tyrosine	X	X	X
Valine	X	X	X

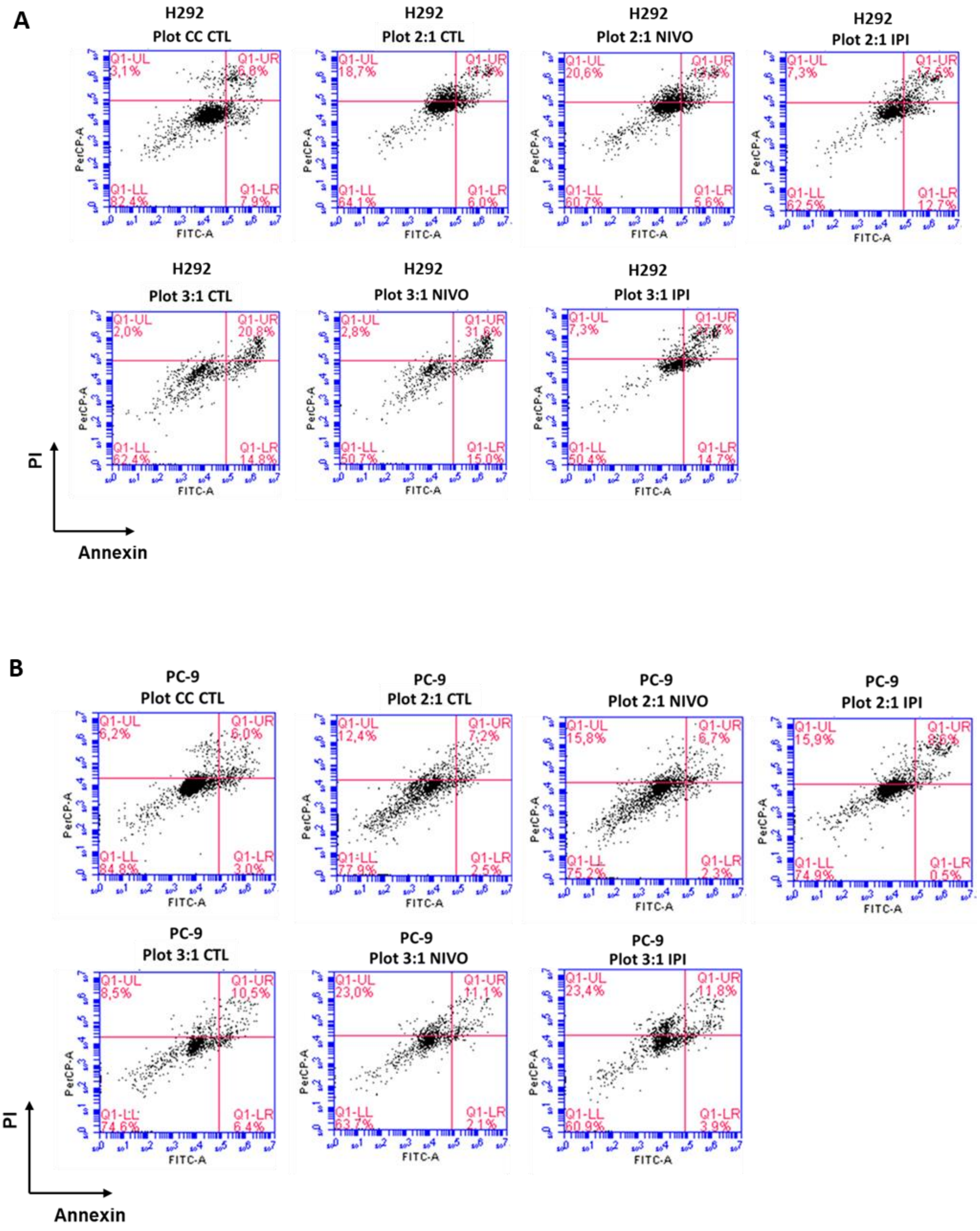

Supplementary Figure S1. The presence of TCD8+, Nivolumab and Ipilimumab affect viability of H292 and PC-9 cells. NSCLC cells were co-cultured with TCD8+ in different TCD8+: Cancer cell ratios. The cells were cultured in control (CTL), Nivolumab (NIVO) and Ipilimumab (IPI) conditions and cell death was evaluated by flow cytometry (N=3). In this figure, the cytometry plots obtained for 2:1 and 3:1 ratios are presented, with the staining of NSCLC cells with Annexin and PI (Propidium iodide). (A) Representative flow cytometry plots of H292 cells, including the untreated condition (CC: cancer cells) and the conditions that promoted significant alterations in cell death (2:1 and 3:1 ratios). (B) Representative flow cytometry plots of PC-9 cells, including the untreated condition (CC: cancer cells) and the conditions that promoted significant alterations in cell death (2:1 and 3:1 ratios).

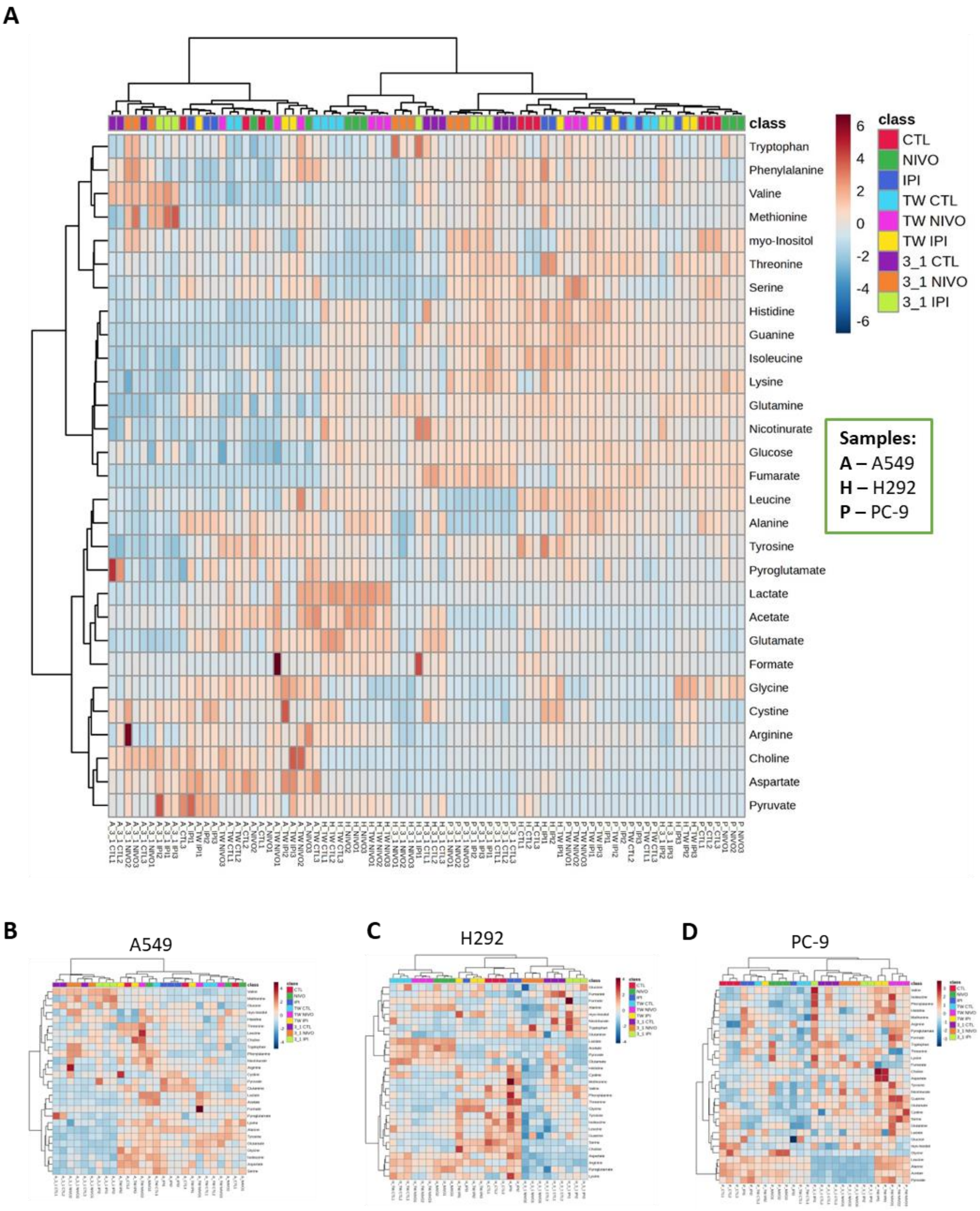

Supplementary Figure S2. The TME exometabolome is affected by TCD8^{+} and ICI treatment. The cell culture media were analyzed by Nuclear Magnetic Resonance (NMR) in order to characterize the TME exometabolome and detect metabolic variations between the experimental conditions (N=3). Detailed heatmaps showing the concentrations of extracellular metabolites in the three cell lines pooled together **(A)** and within A549 **(B)**, H292 **(C)** and PC-9 **(D)** cell lines individually. Concentration data in the heatmaps represent the values obtained for each replicate of the experimental conditions. Heatmaps colors code represent the relative fold change of each metabolite between classes, being red and blue colors increased or decreased levels, respectively. Euclidean distance measure and the Ward

cluster algorithm were used for the heatmaps analysis, using MetaboloAnalyst 5.0 software. Sample names indicate their respective cell line (A – A549, H – H292, P – PC-9) and the condition studied (CTL, NIVO, IPI, TW CTL, TW NIVO, TW IPI, 3:1 CTL, 3:1 NIVO, 3:1 IPI). TW: TransWell conditions (indirect co-cultures).
